# Effect of hyperthermic intraperitoneal chemotherapy in combination with cytoreductive surgery on the prognosis of patients with colorectal cancer peritoneal metastasis: a systematic review and meta-analysis

**DOI:** 10.1186/s12957-022-02666-3

**Published:** 2022-06-14

**Authors:** Ji Li, An-Ran Wang, Xiao-Dong Chen, Yu-Xin Zhang, Hong Pan, Shi-Qiang Li

**Affiliations:** 1 General Surgery, Chongqing Western Hospital, Chongqing, 400051 China; 2 Department of Radiology, the First Affiliated Hospital of Chongqing Medical University, Chongqing, 400042 China

**Keywords:** Colorectal cancer, Peritoneal metastasis, Cytoreductive surgery, Hyperthermic intraperitoneal chemotherapy, Meta-analysis

## Abstract

**Background:**

Peritoneal metastasis often occurs in patients with colorectal cancer peritoneal metastasis, and the prognosis is poor. A large body of evidence highlights the beneficial effects of cytoreductive surgery (CRS) and hyperthermic intraperitoneal chemotherapy (HIPEC) on survival, but to date, there is little consensus on the optimal treatment strategy for patients with colorectal cancer peritoneal metastasis. The purpose of this study is to evaluate the impact of CRS + HIPEC on survival and provide reference for the treatment of patients with colorectal cancer peritoneal metastasis.

**Methods:**

This systematic review and meta-analysis is reported in accordance with the Preferred Reporting Items for Systematic Reviews and Meta-Analyses (PRISMA) statement. The PubMed, Embase, Cochrane, Web of Knowledge, and ClinicalTrials.gov databases were screened from inception of the review to March 11, 2022. Ten studies were included in qualitative and quantitative analysis.

**Results:**

A total of 3200 patients were enrolled in the study, including 788 patients in the CRS and HIPEC groups and 2412 patients in the control group, of which 3 were randomized controlled trials and 7 were cohort studies. The 3 randomized controlled studies were of high quality, and the quality scores of the 7 cohort studies were all 7 or above, indicating high quality. The results showed that the OS of CRS + HIPEC group was higher than that of control group (*HR*: 0.53, 95% *CI*: 0.38–0.73; *P* < 0.00001, *I*^2^ = 82.9%); the heterogeneity of the studies was large. The subgroup analysis showed that the OS of CRS and HIPEC group was higher than that of PC group (*HR*: 0.37, 95% *CI*: 0.30–0.47; *P* = 0.215, *I*^2^ = 31%) and higher than that in CRS group (*HR*: 0.73, 95% *CI*: 0.49–1.07; *P* = 0.163, *I*^2^ = 44.8%); the heterogeneity of the studies was low. In the OPEN group, the OS of THE CRS and HIPEC groups was higher than that in the control group (*HR*: 0.51, 95% *CI*: 0.38–0.70; *P* = 0.353, *I*^2^ = 3.9%); OPEN group showed lower heterogeneity. The OS of 60–100-min group was higher than that in the control group (*HR*: 0.65, 95% *CI*: 0.49–0.88; *P* = 0.172, *I*^2^ = 37.4%); the heterogeneity of the studies was low. Sensitivity analysis showed that there was no significant difference in the results of the combined analysis after each study was deleted. The results of publication bias showed that the *P*-value of Egger and Begg tests was 0.078 > 0.05, indicating that there is no publication bias.

**Conclusions:**

CRS + HIPEC can improve the survival rate of patients with colorectal cancer peritoneal metastasis

## Introduction

Colorectal cancer is responsible for close to 10% of cancer diagnoses and deaths throughout the world, with about 2 million new diagnoses per year [1]. Of these, between 20 and 25% of patients have advanced cancer, with the same numbers developing metastases after surgery [2]. Metastasis to the peritoneum and liver is common [3, 4]. Peritoneal metastases (PM) usually present with relatively nonspecific symptoms and are thus often only detected at advanced stages; thus, PM are associated with poor outcomes [5]. If untreated, such patients typically do not live longer than a year [6]. Systemic treatment for PM has limited success, often only increasing the median survival from 12 to 16 months [7]. In this context, cytoreductive surgery (CRS) combined with hyperthermic intraperitoneal chemotherapy (HIPEC) has been found to be successful for treating colorectal cancer accompanied by PM [8], and this combination, although initially developed for treating pseudomyxoma peritonei, is now accepted as a standard surgical treatment for all malignancies of the peritoneal surface regardless of their origin [9]. Patients have been found to respond well to this treatment, with median overall survival (OS) rates increasing to between 20 and 63 months and 5-year OS rates of 23–52% [10, 11]. Specific outcomes are associated with various factors representing the severity of the disease, including the peritoneal cancer index (PCI), the completeness of cytoreduction (CC), and tumor histopathology [12]. The success of CRS + HIPEC is dependent on the careful selection of suitable patients (e.g., *PCI* < 20), in whom the combined therapy has been reported to be better than the best current systemic chemotherapies [13]. However, the indications for CRS + HIPEC used in different centers vary considerably. Eastern Cooperative Oncology Group or World Health Organization indices > 2, together with the presence of critical comorbidities, such as severe cardiopulmonary or renal failure, usually represent contraindications for patient selection [14]. Age is also a factor, although there are no specific contraindications, and the presence of liver metastases complicates the issue. Several recent reports have indicated the effectiveness of liver metastasis resection in improving survival without causing additional morbidity [15, 16], although the optimal number of liver metastases influencing the effectiveness of CRS + HIPEC remains controversial [17]. However, there are limited data on the suitable treatment of patients with PM. Currently, the standard treatment is a combination of systemic and palliative therapy, and there is little consensus on the optimal treatment for these patients. Thus, the objective of the current systematic review and meta-analysis was to review and analyze studies on the use and effectiveness of CRS + HIPEC for treating patients with colorectal cancer and PM and to provide a reference for clinical practice.

## Methods

### Search strategy

This study conforms with the Preferred Reporting Items for Systematic Reviews and Meta-Analyses (PRISMA) statement. The protocol for this systematic review was registered on INPLASY (INPLASY202230093) and is available in full on inplasy.com (10.37766/inplasy2022.3.0093).

The PubMed, Embase, Cochrane, Web of Knowledge, and ClinicalTrials.gov databases were searched from inception to March 11, 2022. Articles in all languages were searched. The complete search terms used for PubMed were as follows: ((((((((((((Hyperthermic Intraperitoneal Chemotherapy [Title/Abstract]) OR (Chemotherapy, Hyperthermic Intraperitoneal[Title/Abstract])) OR (Intraperitoneal Chemotherapy, Hyperthermic[Title/Abstract])) OR (HIPEC[Title/Abstract])) OR (Hot Chemotherapy[Title/Abstract])) OR (Chemotherapy, Hot[Title/Abstract])) OR (Intraperitoneal Hyperthermic Chemotherapy[Title/Abstract])) OR (Chemotherapy, Intraperitoneal Hyperthermic[Title/Abstract])) OR (Chemotherapy, Intraperitoneal Hyperthermic [Title/Abstract])) OR (Intraperitoneal Hyperthermic Chemotherapies[Title/Abstract])) AND ((((((((((((((((Colorectal Neoplasms[Title/Abstract]) OR (Colorectal Neoplasm[Title/Abstract])) OR (Neoplasm, Colorectal[Title/Abstract])) OR (Neoplasms, Colorectal[Title/Abstract])) OR (Colorectal Tumors [Title/Abstract])) OR (Colorectal Tumor[Title/Abstract])) OR (Tumor, Colorectal[Title/Abstract])) OR (Tumors, Colorectal[Title/Abstract])) OR (Colorectal Cancer [Title/Abstract])) OR (Cancer, Colorectal[Title/Abstract])) OR (Cancers, Colorectal[Title/Abstract])) OR (Colorectal Cancers [Title/Abstract])) OR (Colorectal Carcinoma[Title/Abstract])) OR (Carcinoma, Colorectal[Title/Abstract])) OR (Carcinomas, Colorectal[Title/Abstract])) OR (Colorectal Carcinomas[Title/Abstract]))) AND ((cytoreductive surgery[Title/Abstract]) OR (CRS [Title/Abstract]))). All potentially eligible studies were considered, regardless of primary outcomes or language.

### Inclusion criteria

A population (P), intervention (I), comparator (C), outcome (O), and study design (S) (PICOS) framework was used to describe the eligibility of studies. Specifically, the criteria below were included:

- Population (P): patients with colorectal cancer with PM
- Intervention (I): complete CRS + HIPEC
- Comparison (C): patients undergoing surgery or any other systemic palliative therapy
- Outcomes (O): patient survival outcomes
- Study design (S): randomized controlled trials, case-control studies, or cohort studies

### Exclusion criteria

Articles that did not contain survival data were excluded, as were studies investigating CRS + HIPEC in primary tumors other than colorectal cancer. Similarly, composite studies that included patients with colorectal cancer or other malignancies but did not report isolated results were considered ineligible.

### Data extraction and quality assessment

The literature screening was conducted by two researchers (JL and ARW) independently, through reading the subject, selecting the standard subject, and subsequently reading the abstract and the full text. For randomized controlled studies, the two researchers cross-estimated the quality of the studies using the Jadad scale, including random allocation, randomized hiding, double-blind method setting, and exit and loss to follow-up (score out of 7 points: 1−3 for inferior quality and 4–7 points for good quality), while the evaluation of methodological quality used the method recommended by the Cochrane Review handbook. The Newcastle-Ottawa scale (NOS) was used for quality assessment of case-control and cohort studies; this includes eight items divided into three areas, namely, population selection, comparability, and exposure or outcome evaluation, using a scale of 0–9 points with scores above 5 rated as high quality [18]. Two researchers independently recorded the necessary information from the publications, including details of the first author, publication date, number of subjects, time of enrollment, type of study, treatment details of the control group, and the hazard ratios (HRs) for the experimental and control groups and their 95% confidence intervals (CIs). Any differences between the two researchers were decided by discussion with a third researcher (SQL).

### Statistical analysis

The HR and 95% CI values in both groups were pooled and analyzed. If the HR and its 95% CI could not be extracted, data were extracted from survival curves using Engauge Digitizer software and converted. Inter-study heterogeneity was evaluated using the *I*^2^ statistic and Cochran’s Q test, with cutoff values of 25%, 50%, and 75% considered as low, moderate, and high, respectively [19]. Sensitivity analysis was performed in relation to the assessed effect sizes and heterogeneity of the studies. The risk of publication bias was assessed using funnel plots, with the asymmetry of the plot indicating potential bias; asymmetry was analyzed by Egger’s and Begg’s tests. Intercept significances were assessed using *t*-tests (*P* < 0.05).

## Results

### Features of the included studies

**Fig. 1:**
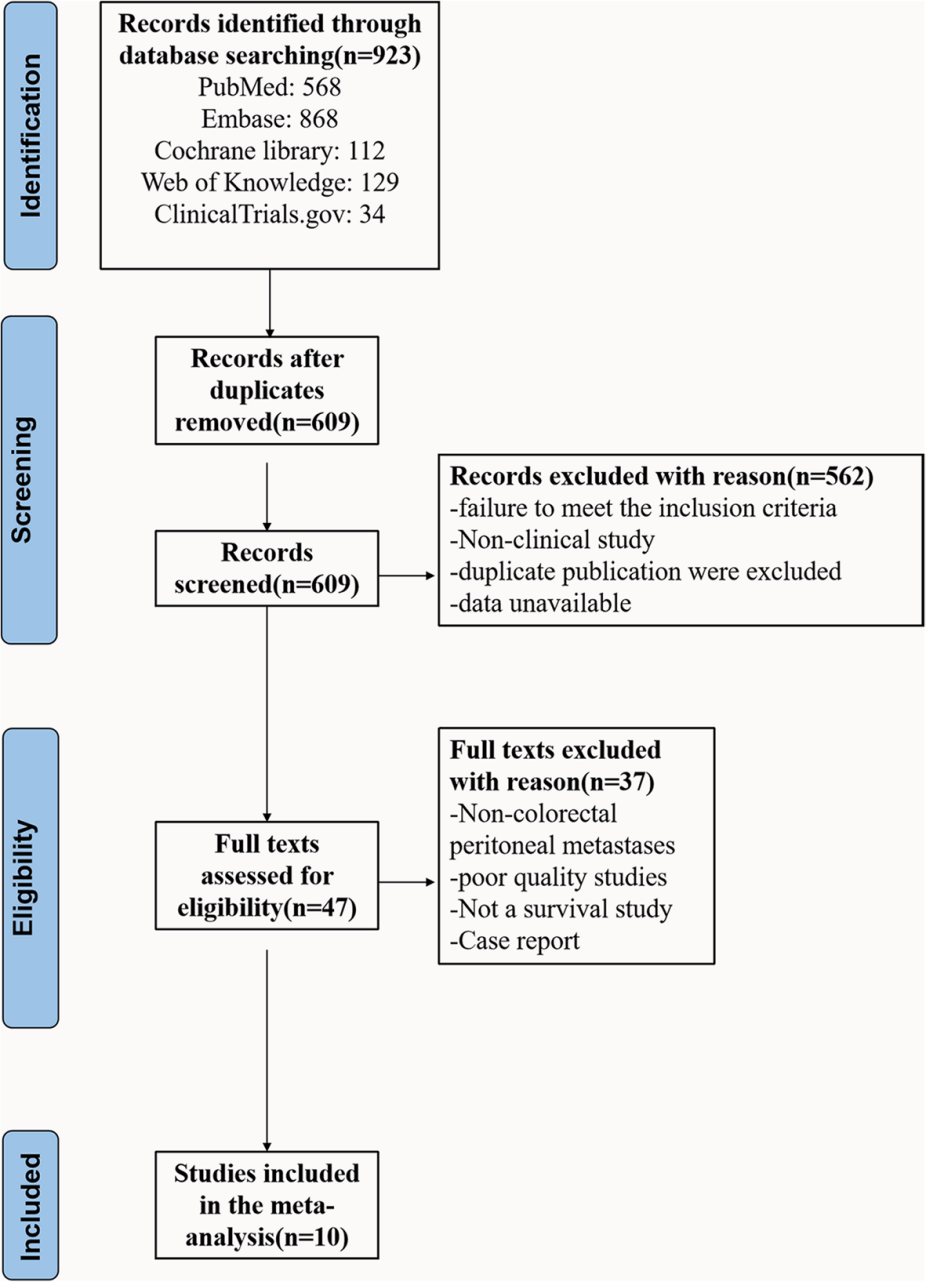
Flow chart of screening strategy for included studies

In all, 923 studies were initially identified. Duplicates between databases were removed, leaving 609 studies that were then screened in terms of titles and abstracts. A further 562 papers were subsequently excluded for not meeting the inclusion criteria, leaving 47 studies. Of these, a further 37 studies were excluded after examination of the full texts for the following reasons: (1) non-colorectal peritoneal metastases; (2) poor-quality studies; (3) not a survival study; and (4) case report. Finally, 10 studies [20–29] were included in the meta-analysis (Fig. 1). These included 3200 patients, with 788 patients in the CRS and HIPEC groups and 2412 patients in the control group. Three studies [22, 27, 29] were randomized controlled trials, and seven [20, 21, 23–26, 28] were cohort studies. The details of the included studies are summarized in Table 1.

**Table 1:** Main characteristics of all studies included in the meta-analysis

Author	Year	Country	Enrollment	Type	HIPEC group (***n***)	Control group (***n***)	HIPEC characteristics		Control characteristics	HR 95% ***CI***
							Technique	Time (min)		
Franko	2010	American	2001–2007	Cohort	67	38	Close	100	PC	0.42 (0.19–0.91)
Cashin	2012	Sweden	1996–2010	Cohort	69	57	Open	30	CRS + SPIC	0.60 (0.36–0.99)
Chen	2020	China	2008–2016	Randomized	14	14	Close	90	HIPEC + dCRS	0.98 (0.57–1.32)
Razenberg	2015	Netherlands	2005–2012	Cohort	297	1980	NR	NR	PC	0.36 (0.36–0.43)
Huang	2014	China	2005–2013	Cohort	33	29	Close	90	CRS	0.47 (0.25–0.85)
Gervais	2013	Canada	2004–2011	Cohort	25	15	Close	30	PC	0.21 (0.12–0.52)
Elias	2009	France	1998–2003	Cohort	48	48	Open	30	PC	0.35 (0.19–0.64)
Verwaal	2008	Netherlands	1998–2001	Randomized	54	51	Open	90	PC	0.57 (0.36–0.93)
Baratti	2020	Italy	2012–2018	Cohort	48	48	Close	60	CRS	0.73 (0.47–1.15)
Quénet	2021	France	2008–2014	Randomized	133	132	NR	30	CRS	0.99 (0.62–1.57)

*SPIC* Sequential postoperative intraperitoneal chemotherapy, *CRS* Cytoreductive surgery, *PC* Palliative chemotherapy, *dCRS* Delayed cytoreductive surgery; open, the open Coliseum technique; close, the close Coliseum technique; *NR*, not reported

### Quality assessment of the included studies

The Jadad scale was used to assess the quality of the randomized controlled trials, with scores between 1 and 3 indicating inferior quality and scores between 4 and 7 representing high quality. This evaluation showed that three studies were of high quality (Tables 2 and 3) The NOS, with scores between 5 and 9 indicating good quality, was used for the assessment of case-control and cohort studies and showed that the scores of all seven studies were above 7, indicative of high quality.

**Table 2:** Quality assessment of included trials using the Jadad scale

*First author*	*Type*	*Random allocation*	*Randomized hiding*	*Double-blind method setting*	*Exit and loss to follow-up*	*Score*
Chen	RCT	2	2	2	0	6
Verwaal	RCT	1	1	1	1	4
Quénet	RCT	2	2	2	1	7

*RCT* Randomized controlled trial

**Table 3:** Results of quality assessment using the Newcastle-Ottawa scale for cohort studies

Study	Selection				Comparability	Outcome			Score
	Representativeness of the exposed cohort	Selection of the nonexposed cohort	Ascertainment of exposure	Demonstration that outcome of interest was not present at start of study	Comparability of cohorts on the basis of the design or analysis	Assessment of outcome	Was follow-up long enough for outcomes to occur	Adequacy of follow-up of cohorts	
Franko	●	●	●	●	●	●	●	●	8
Cashin	●	●	●	●		●	●	●	7
Razenberg	●	●	●	●		●	●	●	7
Huang	●	●	●	●	●	●	●	●	8
Gervais	●	●	●	●		●	●	●	7
Elias	●	●	●	●	●	●	●	●	8
Baratti	●	●	●	1	●	●	●	●	8

### Meta-analysis

**Fig. 2:**
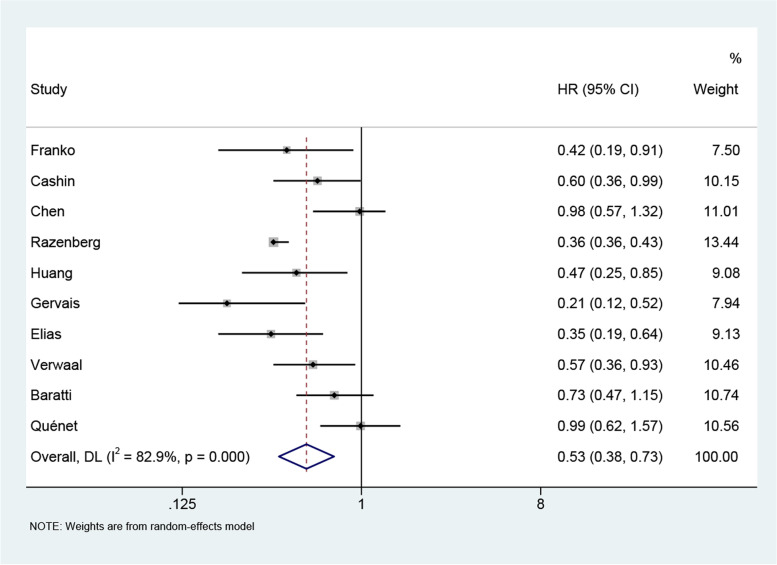
Meta-analysis of overall survival (OS) of patients with colorectal cancer peritoneal metastasis treated with CRS + HIPEC versus control group

**Fig. 3:**
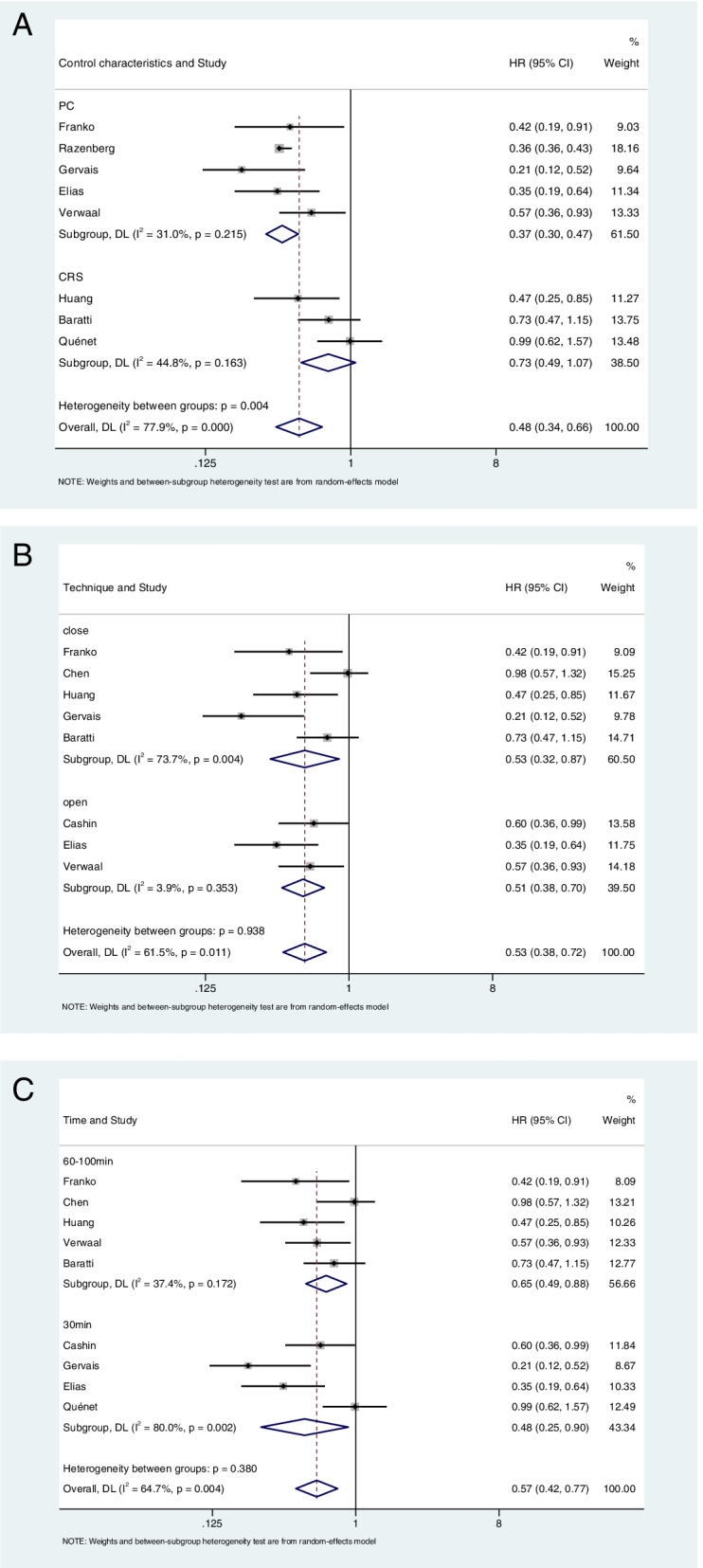
Subgroup analysis of colorectal cancer peritoneal metastasis treated with CRS + HIPEC and control group. **A** Subgroup analysis of different treatment regimens. **B** Subgroup analysis of different treatment devices. **C** Subgroup analysis of different HIPEC time. PC palliative chemotherapy, open the open Coliseum technique, close the close Coliseum technique

It was found that the OS of the CRS + HIPEC group was higher than that of the control group (*HR*: 0.53, 95% *CI*: 0.38–0.73; *P* < 0.00001, *I*^2^ = 82.9%) (Fig. 2). Due to the large heterogeneity of the study, we then performed relevant subgroup analysis. This indicated that the OS of the CRS and HIPEC group was superior to that of the PC group (*HR*: 0.37, 95% *CI*: 0.30–0.47; *P* = 0.215, *I*^2^ = 31%) and higher than that of the CRS group (*HR*: 0.73, 95% *CI*: 0.49–1.07; *P* = 0.163, *I*^2^ = 44.8%) (Fig. 3A). The heterogeneity of the subgroups was low. We then divided the experimental groups into an OPEN group and a CLOSE group [30] according to the different HIPEC devices used. In the OPEN group, the OS rates of the CRS and HIPEC groups were higher than in the control group (*HR*: 0.51, 95% *CI*: 0.38–0.70; *P* = 0.353, *I*^2^ = 3.9%), while in the CLOSE group, the OS rates of the experimental group were higher (*HR*: 0.53, 95% *CI*: 0.32–0.87; *P* = 0.004, *I*^2^ = 73.7%). In addition, the OPEN group showed lower heterogeneity (Fig. 3B). After division into various subgroups based on the duration of HIPEC treatment, the 30-min group (*HR*: 0.48, 95% *CI*: 0.25 –0.90; *P* = 0.002, *I*^2^ = 80%) and the 60–100-min group (*HR*: 0.65, 95% *CI*: 0.49–0.88; *P* = 0.172, *I*^2^ = 37.4%) had longer OS than the control group, while the heterogeneity was lower in the 60–100-min group (Fig. 3C).

### Assessment of publication bias

**Fig. 4:**
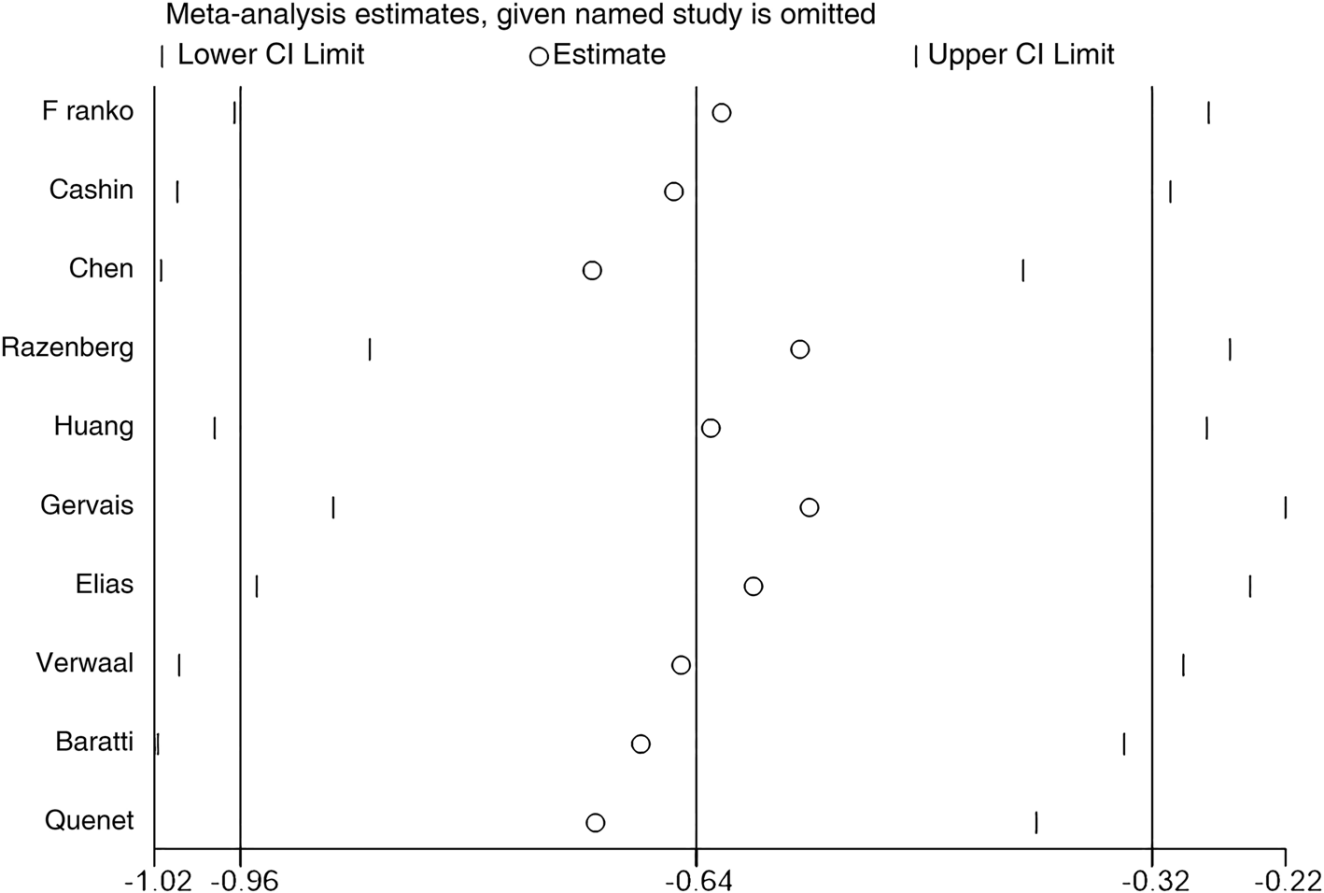
Sensitivity analysis

**Fig. 5:**
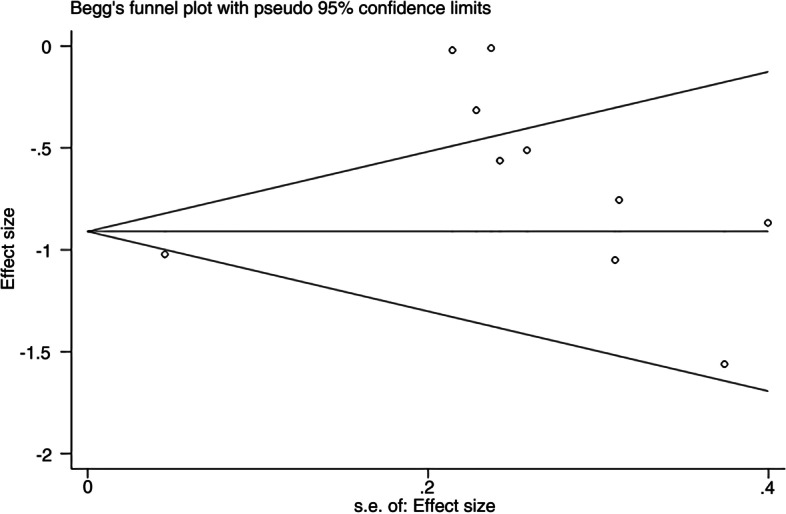
Begg funnel plot for publication bias test

Sensitivity analysis indicated no significant differences in the results of the combined analysis after the deletion of individual studies, showing that the overall results were reliable (Fig. 4). Assessment of publication bias showed that the *P*-values of the Egger and Begg tests were 0.078 > 0.05. No obvious asymmetry was seen in the Begg funnel plot, indicating an absence of publication bias (Fig. 5).

## Discussion

The objective of the current systematic review and meta-analysis was to investigate the outcomes of CRS + HIPEC in patients with colorectal cancer and PM. The analysis assessed outcomes in terms of OS, combining HR and 95% CI for the trial and control groups. It was found that the combined use of CRS + HIPEC was superior to both PC and CRS in extending the OS of these patients. These data offer evidence for the effectiveness of CRS + HIPEC for treating patients with colorectal cancer and PM, as well as laying a foundation for future prospective studies in these patients.

The reduction of tumor dimensions has long been recognized as critical in the response of the cancer to therapeutic intervention. CRS involves extensive peritoneal and visceral excision to remove all visible tumor foci, with the goal of minimizing tumor size [31]. Pharmacokinetics has shown that intraperitoneal drug administration is more effective than intravenous administration as the drugs are able to interact directly with the tumor cells while reducing the systemic levels and thus the potential adverse effects of the drugs. HIPEC permits the delivery of high drug concentrations, and their cytotoxicity to tumor cells is increased by hyperthermia [32]. This explains to some extent why the therapeutic effect of CRS and HIPEC is superior to other treatment regimens. Various factors have been found to affect the clinical efficacy of CRS + HIPEC. These include the PCI and CC, as well as the presence of serious adverse events, the status of lymph nodes, the use of systemic chemotherapeutic drugs, and peritoneal carcinomatosis, whether synchronous or metachronous. The Sugarbaker PCI score, ranging from 0 to 39, is the most commonly used PCI standard [33], with scores of 0–19 representing LPCI and those over 20, HPCI [34, 35]. Sugarbaker et al. [36] also reported 5-year OS rates of 50%, 20%, and 0% for patients with scores below 10, between 11 and 20, and over 20, respectively. In terms of CC scores, patients with CC0 experienced better survival outcomes than patients with scores between 1 and 3, with median OS values of 33.0 months and 10.0 months, respectively [37, 38]. However, to eliminate the tumor completely, extensive resection often involving a number of organs and regions of the abdomen is usually required. This may lead to increased blood and fluid loss, disruption of the hemodynamic balance, and an increased likelihood of serious adverse events [39]. In such cases, perioperative morbidity has been found to range between 14.8 and 57.0%, and mortality rates may increase to 12.0% [40]. Two multicenter studies by Glehen et al. [37] and Elias et al. [41] observed the perioperative mortality rates of 4% and 3%, respectively. An additional issue is that the CRS + HIPEC combination has an extended learning curve, which has negatively influenced the clinical popularity of the method [42, 43]. Several studies are currently investigating the factors affecting the posttreatment complications of CRS + HIPEC, aiming to reduce these as far as possible. Rotolo et al. [44] observed that the presence of low skeletal muscle mass at diagnosis influences the development of postoperative complications after CRS in patients with colorectal cancer and PM. Morgan et al. [45] reported that mutation of the RAS gene independently predicted early tumor recurrence after CRS + HIPEC, suggesting that this could be used for the identification of patients who may not benefit from the procedure.

Although we have demonstrated that CRS + HIPEC resulted in a better prognosis for patients with colorectal cancer and PM, the study still has limitations. First, only 10 studies were included, most of which were cohort studies with only three being randomized controlled trials [22, 27, 29]. In terms of subgroup analysis, only three studies compared CRS, and the conclusions drawn from these studies are thus based on limited evidence. Similarly, in the subgroup analysis based on the HIPEC device and treatment duration, although CRS + HIPEC showed better prognosis and lower heterogeneity in the OPEN and 60–100-min groups, the included studies were also limited, and the optimal CRS + HIPEC regimen was not further explored. In terms of publication bias, both the Begg and Egger tests have good sensitivity only when more than 20 studies are included [46], resulting in a low sensitivity result for publication bias. Secondly, when HR and 95% CI values were not provided in included studies, we extracted data through Engauge Digitizer software, which would inevitably lead to some error. Finally, we observed that the HR values of the two included high-quality randomized controlled trials [22, 29] were 0.98 (95% *CI*: 0.57–1.32) and 0.99 (95% *CI*: 0.62–1.57), respectively, which did not show satisfactory HR values. However, another randomized controlled trial [28] had an HR value of 0.57 (95% *CI*: 0.36–0.93). Thus, more analysis of randomized controlled trials is required in the future. All in all, the quality of the included studies was high, which provides evidence supporting the treatment of PM in patients with colorectal cancer by CRS + HIPEC, although further studies are required for verification.

## Data Availability

The datasets used and/or analyzed during the current study are available from the corresponding author on reasonable request.
